# The function and mechanism of COX-2 in angiogenesis of gastric cancer cells

**DOI:** 10.1186/1756-9966-30-13

**Published:** 2011-01-25

**Authors:** Liping Yao, Fei Liu, Liu Hong, Li Sun, Shuhui Liang, Kaichun Wu, Daiming Fan

**Affiliations:** 1 State Key Laboratory of Cancer Biology and Xijing Hospital of Digestive Diseases, Fourth Military Medical University, 15 West Changle Road, Xi'an, 710032, PR China

## Abstract

**Background:**

Here we aimed to investigate the effect of COX-2 siRNA on proliferation and angiogenesis of gastric cancer cells.

**Methods:**

The gastric cancer cell line SGC7901 was transfected with COX-2 siRNA, then the growth and angiogenesis of cells were detected by in vitro and in vivo assay. Human microarray, RT-PCR and western blot were used to identify differentially expressed angiogenesis-related molecules in cells with decreased expression of COX-2.

**Results:**

Down-regulation of COX-2 could significantly inhibit the in vitro and in vivo growth of gastric cancer cells, and suppress the migration and tube formation of human umbilical vein endothelial cells. Totally 23 angiogenesis-related molecules were found involved in COX-2-induced angiogenesis suppression. The results of RT-PCR and western blot showed that down-regulation of COX-2 might inhibit VEGF, Flt-1, Flk-1/KDR, angiopoietin-1, tie-2, MMP2 and OPN.

**Conclusions:**

COX-2 might mediate tumor angiogenesis and growth, and could be considered as a target for gastric cancer therapy.

## Background

Gastric cancer is the second leading cause of cancer associated death in the world, particularly in Asian countries. The treatment outcome of this common malignancy is still not satisfactory and various chemotherapeutic attempts in an adjuvant setting have failed to improve the survival rate in gastric cancer. Recently, angiogenesis has been found related to hematogenous recurrence and poor prognosis in gastric cancer [1]. Angiogenesis is the growth of new vessels from existing vasculature. A balance of angiogenic and angiostatic growth factors tightly controls physiological angiogenesis. Tipping of this balance towards a pro-angiogenic environment is termed the 'angiogenic switch' and occurs in situations such as tissue hypoxia, inflammation or neoplasia [2].

COX-2, a COX isoenzyme catalyzing the production of prostaglandins, has been observed in most gastric cancer tissues compared with the accompanying normal mucosa. Studies in different cancers have suggested a relationship between COX-2 and increased pro-angiogenic growth factors, in particular VEGF [3]. COX-2 is thought to promote angiogenesis and so drive the malignant phenotype. Overexpression of COX-2 might contribute to angiogenesis of gastric cancer [4]. However, the potential mechanism underlying the role of COX-2 in angiogenesis remains unclear.

Here we have demonstrated novel observations that COX-2 might play important roles in angiogenesis of gastric cancer through regulation of VEGF, Flt-1, Flk-1/KDR, angiopoietin-1, tie-2, MMP2 and OPN.

## Methods

### Cell culture

Human gastric cancer cell line SGC7901 was cultivated in Dulbecco's modified Eagle's medium supplemented with 10% heat-inactivated fetal calf serum, penicillin (100 U/ml) and streptomycin (100 μg/ml), in a CO_2 _incubator (Forma Scientific) [5]. Human umbilical vein endothelial cells (HUVEC-12; ATCC, Manassas, VA) were grown in Kaighn's modification of Ham's F12 medium (ATCC) with 2 mM Lglutamine, 1.5 g/l sodium bicarbonate, 0.1 mg/ml heparin, 0.03 mg/ml endothelial cell growth supplement and 10% FBS.

### Plasmid construction and transfection

The siRNA oligos for COX-2 were designed according to previous report. Target sequences were aligned to the human genome database in a BLAST search to ensure that the choosing sequences were not highly homologous with other genes. For oligo-1, S: 5'-tttgcatcgatgtcaccatagaacatctatggtgacatcgatgcttttt-3', AS: 5'-ctagaaaaagcatcgatgtcacc atagatgttctatggtgacatcgatg-3' For annealing to form DNA duplexes, 100 μM of each S and AS oligos was used. The duplexes were diluted and then ligated with mU6pro vector which previously digested by the Bbs I/Xba I restriction enzyme and gel purified at room temperature for 30 min. The products were transformed into DH5α competent cells. Ampicillin-resistant colonies were chosen, identified by restriction digestion and further confirmed by DNA sequencing.

SGC7901 cells were planted in six-well plates and cultured in drug-free medium. At 90-95% confluence, cells were washed twice with PBS, grew in 2 ml of DMEM without antibiotics. Using Lipofectamine™ 2000 reagent (Invitrogen, Inc. Carlsbad CA), 2 μg of mU6pro-COX-2siRNA plasmids were transfected into cells according to the manufacturer's instructions. The cells transfected with mU6pro vector alone were served as negative control. Forty-eight hours later, cells were placed in growth medium containing G418 (GIBCO) for clone selection. The expression levels of COX-2 in G418-resistant clones were evaluated by western blot analysis.

### RT-PCR

All of the PCR products were separated on ethidium bromide stained agarose, and visualized with UV as described previously [6].

### Western blot analysis

The western blot was done as described previously. In brief, total cellular proteins were prepared and then quantified by Bradford method [7]. A measure of 80 ug of lysates were electrophoresed in 12% SDS-PAGE and blotted on a nitrocellulose membrane (Immoblin-P, Millipore, Bedford, MA, USA). Membranes were blocked with 5% fat-free milk powder at room temperature and incubated overnight with antibody at 4°C. After three washes for 15 min in PBS-T, the membrane was incubated with the HRP-conjugated goat anti-mouse IgG antibody (Wuhan, Hubei, China) for 1 h at room temperature. The enhanced chemiluminescence (Amersham Life Science, Piscataway, NJ, USA) was added and monitored for the development of color.

### Cell growth assay

Cells were seeded on a 96-well plate at 3 × 10^4 ^cells/well. Each sample had four replicates. The medium was replaced at 2-day intervals. Viable cells were counted by the 3-[4,5-dimethylthiazol-2-yl]- 2,5-diphenyltetrazolium bromide (MTT) assay after 2, 4, 6, and 8 days.

### Tumor growth in nude mice

Female athymic *nu/nu *mice, 5-6 weeks of age, were obtained from FMMU Experimental Animal Co. (Shaanxi, China) and housed in a pathogen-free facility for all of the experiments. The logarithmically growing cells were trypsinized and resuspended in D'Hanks solution, and 5 × 10^6 ^cells in 0.2 ml were injected subcutaneously into the left flank of mice [8]. Experimental and control groups had at least 6 mice each. Tumors were measured twice weekly with microcalipers, and the tumor volume was calculated according to the formula: volume = length × (width^2^)/2.

### Quantification of tumor microvessel density

Tumor microvessel densities (MVD) were quantified by anti-CD31 immunohistochemistry. Briefly, tumor sections from nude mice were cut using a LEICA cryostat and the paraffin sections were mounted on positively charged Superfrost slides and dried overnight. The immunostaining was done according to standardized protocols.

### Tube formation assay

Tube formation assay was performed as described previously (Chia et al, 2010). Briefly, Confluent HUVEC cells were harvested and diluted in DMEM with 10% FBS, which were then seeded on Matrigel-coated 24-well plates. Cell culture medium was then replaced by conditioned medium. After 16 h, Matrigel was fixed, stained with H & E and examined under inverted microscope. The mean tube length in five random fields per well was quantified by computer software.

### Cell migration assay

Briefly, confluent monolayer of HUVEC was cultured with non-growth factor containing media for 12 h before harvesting. Harvested cells were suspended in serum-free DMEM199 and HUVEC cells were seeded onto tissue culture inserts in triplicate. The inserts were removed after 8 h culture and washed with PBS. Non-migrated cells on the upper surface of the inserts were removed by wiping with cotton swabs. The inserts were fixed in neutral buffered formalin solution, stained with hematoxylin and eosin (H & E) and mounted on microscope slides. HUVEC migration was quantitated by counting the number of cells in three random fields (!200) per insert.

### cDNA microarray analysis

The gene expression was compared between SGC7901-siRNA and SGC7901-vector cells for three times [9]. RNA was extracted from 80-90% confluent cells using Trizol and purified with RNeasy spin columns (Qiagen, Valencia, CA) according to the manufacturers' instructions. Quality of the RNA was ensured before labeling by analyzing 20 to 50 ng of each sample using the RNA 6000 NanoAssay and a Bioanalyzer 2100 (Agilent, Palo Alto, CA). Samples with a peak ratio of 1.8 to 2.0 were considered suitable for labeling. Cy3- or Cy5-labeled cDNA was generated and the Cy3/Cy5 single-stranded cDNA/cot1 DNA pellet was resuspended in hybridization buffer, then the hybridization mix was applied to GEArray Q Series Human Angiogenesis Gene Array. The ratios of gene expression were considered to be significant if they were 2 or 0.5 in at least two independent experiments. Genes were assigned to functional families based on information from LocusLink and PubMed.

### Statistical analysis

Data were presented as mean ± standard deviation (S.D.) unless otherwise specified. Comparisons between groups were made using the Student-Newman-Keuls test or the Kruskal-Wallis test. All data were analyzed using the SPSS software package (SPSS Inc, Chicago, USA). A value of P < 0.05 was considered significant.

## Results

### Down-regulation of COX-2 inhibited the growth and tumorigenecity of gastric cancer cells

**Figure 1:**
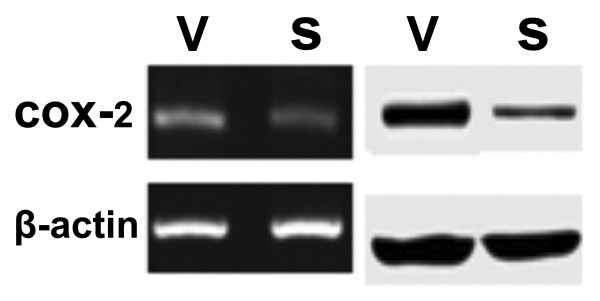
**RT-PCR (left) and western blot analysis (right) of COX-2 in the vector transfectants SGC7901-V (V) and the siRNA transfectants SGC7901-siRNA (S)**. ß-actin was used as loading control.

**Figure 2:**
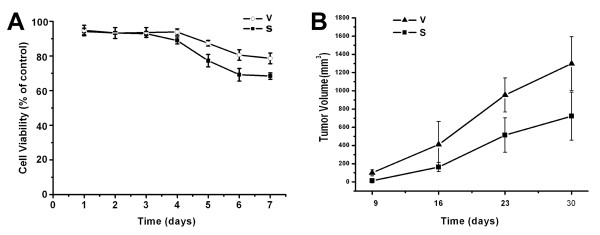
**Down-regulation of COX-2 suppressed growth of gastric cancer cells in vitro and in vivo**. A, The growth rate of the cells was detected using MTT assay as described in "Materials and Methods". The value shown was the mean of three determinations. B, tumorigenicity of the cells in BALB/c nu/nu mice was detected. Each group had at least 6 mice. The volumes of tumors were monitored at the indicated time.

As **Figure**1 showed, SGC7901 cells were transfected and then one resistant clone (SGC7901-siRNA) with significantly decreased COX-2 expression and one vector transfected control clone (SGC7901-vector) were selected. The results of MTT assay showed that down-regulation of COX-2 might significantly decrease the proliferation of SGC7901 cells (**Figure**2A). As shown in **Figure**2B, down-regulation of COX-2 might inhibit the malignant growth of SGC7901 cells in vivo.

### Down-regulation of COX-2 inhibited angiogenesis of gastric cancer cells

**Figure 3:**
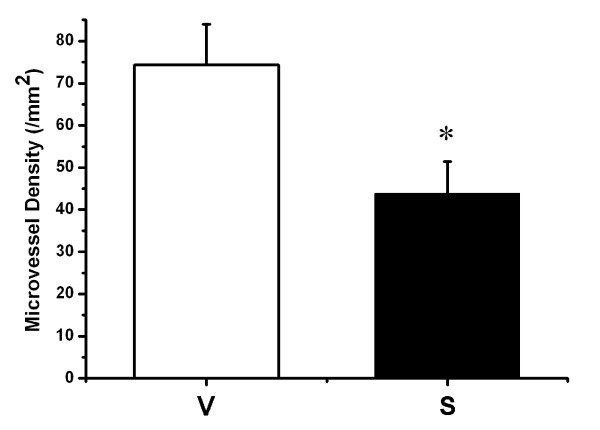
**Effects of COX-2 on tumor angiogenesis**. The tumor microvessel densities (means) in sections from tumors formed by the vector transfectants SGC7901-V (V) and the siRNA transfectants SGC7901-siRNA (S). Tumor samples were immunostained with antibodies against CD31. Mean ± SD, n = 3. *, P < 0.05 VS. control.

**Figure 4:**
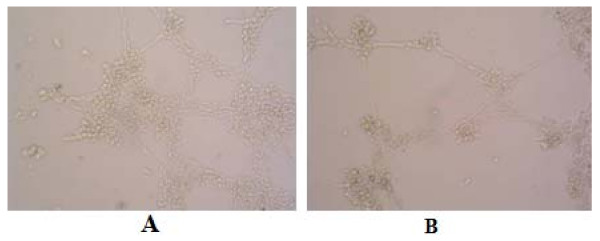
**Effects of conditioned media on HUVEC tube formation. HUVECs were seeded in triplicate on Matrigel-coated 24-well plates, and incubated for 16 h with control SGC7901 medium (A) and COX-2-siRNA medium (B)**.

**Figure 5:**
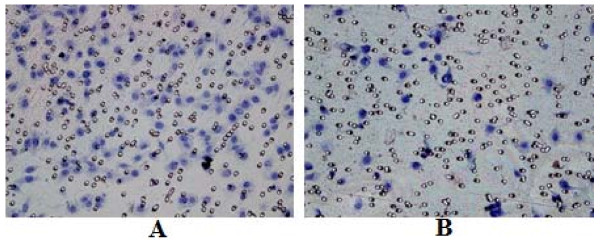
**Effects of conditioned media on HUVEC migration**. Migration assay was performed in a BioCoate Matrigele invasion chamber. The lower chambers were added with control SGC7901 medium (A) and COX-2-siRNA medium (B).

As shown in **Figure**3, the number of endothelial cells within the tumors formed by COX-2-downregulating cells was less than that of tumors formed by control cells. In order to investigate the angiogenic property of COX-2 in endothelial cells, the in vitro tube formation of HUVEC was assessed. As shown in **Figure**4, 5, down-regulation of COX-2 might suppress cell tube formation and migration in HUVEC.

### Effect of COX-2 on angiogenesis related molecules

**Figure 6:**
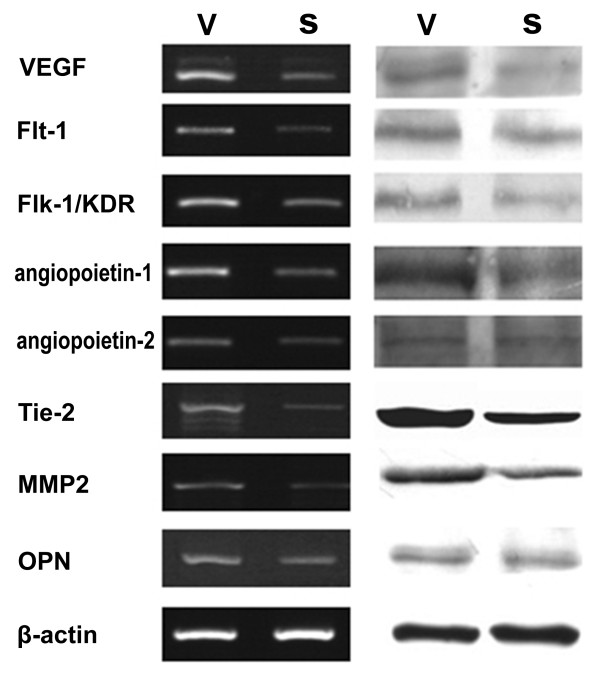
**Expression of VEGF, Flt-1, Flk-1/KDR, angiopoietin-1, angiopoietin-2, tie-2, MMP2 and OPN in the vector transfectants SGC7901-V (V) and the siRNA transfectants SGC7901-siRNA (S) by RT-PCR (left) and Western blot (right)**.

Using cDNA microarray, genes were identified differentially expressed between different transfected SGC7901 cells. Compared with control cells, a total of 23 genes were found to be differentially expressed in COX-2-downregulating cells, including FGF4, PDGF-BB, PDGFRB, PF4, TGFB2, TGFBR1, VEGF, FLT1, FLK 1, angiopoietin-1, angiopoietin-2, Tie2, IFNA1, PRL, PTN, SCYA2, SPARC, TNFSF15, PECAM1, MMP2, SERPINF1, THBS2 and OPN. To confirm the microarray findings, RT-PCR and western blot were undertaken in gastric cancer cells. Down-regulation of COX-2 might inhibit VEGF, Flt-1, Flk-1/KDR, angiopoietin-1, tie-2, MMP2 and OPN (**Figure**6).

## Discussion

Angiogenesis is an essential process required for the growth and metastatic ability of solid tumors. Tumor angiogenesis is the proliferation of a network of blood vessels penetrating into the cancerous growths to supply nutrients and oxygen and remove metabolic waste products from tumors. Tumor angiogenesis is a complex process and involves the tight interplay of tumor cells, endothelial cells, phagocytes and their secreted factors, which may act as promoters or inhibitors of angiogenesis [10]. More than a dozen different proteins (such as VEGF, bFGF, IL8, etc.), as well as several smaller molecules (such as adenosine, PGE, etc.) have been identified as angiogenic factors secreted by tumor cells to mediate angiogenesis [11,12].

Lines of evidence suggest that COX-2 is involved in the steps of gastric carcinogenesis. Increased expression of COX-2 was frequently found in gastric cancer, in which COX-2 expression is correlated with poor prognostic outcome. Up-regulation of cox-2 expression and activity in the ulcer base not only during the acute phase of inflammation but also in the ulcer healing stage and especially in areas of intense tissue repair [13]. It has been found that cyclooxygenase-2 inhibitors have antiproliferative and antiangiogenic activity in several types of human cancer. However, the mechanism of COX-2 in angiogenesis remains unclear.

In this study, the data showed that down-regulation of COX-2 could significantly inhibit the in vitro and in vivo growth of gastric cancer cell line SGC7901, and suppress the migration and tube formation of human umbilical vein endothelial cells, which was consistent with previous report. To our knowledge, we have firstly identified a expression pattern of angiogenesis-related molecules in COX-2-mediated angiogenesis. The results of RT-PCR and western blot showed that down-regulation of COX-2 might inhibit VEGF, Flt-1, KDR, angiopoietin-1, tie-2, MMP2 and OPN.

## Conclusions

In conclusion, COX-2 might mediate tumor angiogenesis and growth, and could be considered as a target for gastric cancer therapy. It was becoming increasingly clear that the signals that govern angiogenesis, functioned in complex regulatory networks rather than simple linear pathways, and that these networks might be wired differently in different cells or tumor types. The precise mechanism by which COX-2 brought about these changes, and which of these changes were primary or secondary ones, remained to be elucidated.

## Competing interests

There is no conflict of interest. The authors declare that they have no competing interests.

## Authors' contributions

Liping Yao, Fei Liu have made substantial contributions to conception and design, acquisition of data, and analysis of data. Liu Hong drafted the manuscript. Li Sun performed the statistical analysis. Shuhui Liang and Kaichun Wu have been involved in revising it critically for important intellectual content. Daiming Fan participated in its design and gave final approval of the version to be published. All authors read and approved the final manuscript.
